# Deep Immunoprofiling of Large-Scale Tuberculosis Dataset at Single Cell Resolution Reveals a CD81^bright^ γδ T Cell Population Associated with Latency

**DOI:** 10.3390/cells13181529

**Published:** 2024-09-12

**Authors:** Mojtaba Shekarkar Azgomi, Giusto Davide Badami, Miriam Di Caro, Bartolo Tamburini, Miriana Fallo, Costanza Dieli, Kiana Ebrahimi, Francesco Dieli, Marco Pio La Manna, Nadia Caccamo

**Affiliations:** 1 Central Laboratory of Advanced Diagnosis and Biomedical Research (CLADIBIOR), Azienda Ospedaliera Universitaria Policlinico (A.O.U.P.) Paolo Giaccone, University of Palermo, 90127 Palermo, Italy; mojtaba.shekarkarazgomi@unipa.it (M.S.A.); giustodavide.badami@unipa.it (G.D.B.); miriam.dicaro01@unipa.it (M.D.C.); bartolo.tamburini@unipa.it (B.T.); miriana.fallo@gmail.com (M.F.); costanza.dieli@unipa.it (C.D.); francesco.dieli@unipa.it (F.D.); marcopio.lamanna@unipa.it (M.P.L.M.); 2 Department of Biomedicine, Neurosciences and Advanced Diagnostic (B.N.D.), University of Palermo, 90127 Palermo, Italy; 3 Department of Health Promotion, Mother and Childcare, Internal Medicine and Medical Specialties, University of Palermo, 90129 Palermo, Italy; 4 Faculté d’Ingénierie et Management de la Santé (ILIS), Université de Lille, 59120 Loos, France; kiana.ebrahimi.etu@univ-lille.fr

**Keywords:** tuberculosis, *Mycobacterium tuberculosis*, latent *Mycobacterium tuberculosis* infection, single-cell RNA sequence, γδ T cells, CD81

## Abstract

Tuberculosis (TB) remains one of the leading causes of death among infectious diseases, with 10.6 million new cases and 1.3 million deaths reported in 2022, according to the most recent WHO report. Early studies have shown an expansion of γδ T cells following TB infection in both experimental models and humans, indicating their abundance among lung lymphocytes and suggesting a role in protective immune responses against *Mycobacterium tuberculosis* (*M. tuberculosis*) infection. In this study, we hypothesized that distinct subsets of γδ T cells are associated with either protection against or disease progression in TB. To explore this, we applied large-scale scRNA-seq and bulk RNA-seq data integration to define the phenotypic and molecular characteristics of peripheral blood γδ T cells. Our analysis identified five unique γδ T subclusters, each with distinct functional profiles. Notably, we identified a unique cluster significantly enriched in the TCR signaling pathway, with high CD81 expression as a conserved marker. This distinct molecular signature suggests a specialized role for this cluster in immune signaling and regulation of immune response against *M. tuberculosis*. Flow cytometry confirmed our in silico results, showing that the mean fluorescence intensity (MFI) values of CD81 expression on γδ T cells were significantly increased in individuals with latent TB infection (TBI) compared to those with active TB (ATB). This finding underscores the importance of CD81 and its associated signaling mechanisms in modulating the activity and function of γδ T cells under TBI conditions, providing insights into potential therapeutic targets for TB management.

## 1. Introduction

A significant global health effect is caused by TB, according to the most recent WHO report on TB, which states that 10.6 million new cases and 1.3 million deaths from TB were estimated by 2022 [1,2]. TBI is a condition where individuals are infected with *M. tuberculosis*, but do not exhibit active TB disease symptoms which is important because it acts as a reservoir for TB, potentially leading to ATB disease in the future if not properly managed. Globally, it is estimated that about a quarter of the world’s population has been infected with *M. tuberculosis*. However, the risk of developing ATB from TBI is higher among people living with HIV and immunocompromised subjects. Addressing TBI is critical in the global strategy to eliminate TB. Enhanced diagnostic methods, effective preventive treatments, and the development of new vaccines are essential steps in managing and reducing the global burden of TBI and preventing the progression to ATB disease. Biomarker discovery is a dynamic and crucial area of TB research, with the potential to transform how TB and TBI are diagnosed and managed. Advances in this field could lead to more effective and timely interventions, ultimately reducing the global burden of TB. Continued research, funding, and global cooperation are essential to realize these advancements and ensure they benefit all populations at risk.

During the early stages of *M. tuberculosis* infection, unconventional T cells function, including γδ T cells, MAIT cells, and lipid-specific CD1-restricted T cells [3]. γδ T cells participate in anti-mycobacterial responses and offer protection against *M. tuberculosis* infection by combining the properties of both innate and adaptive immunity [4,5,6]. Phosphoantigens (PAgs) were first thought to be the primary antigens recognized by the γδ T cell receptor (TCR) since they were discovered to activate γδ T cells [7]. Nonetheless, protective TB immunity is only mediated by a subset of the PAgs-responsive γδ T cells [8].

Early studies have shown expansion of γδ T cells following TB infection both in experimental models and in humans, and demonstrated that γδ T cells abound amongst lung lymphocytes, suggesting they play a role in protective immune responses against *M. tuberculosis* infection [9,10]. There are contrasting data on the relative γδ T cell frequencies and functions in peripheral blood mononuclear cells (PBMCs) of patients with TBI or ATB, compared to healthy controls (HD). Some studies have reported increased frequencies and/or numbers of γδ T cells, while others have shown that γδ T cell numbers remain constant or that they even decrease in the peripheral blood of TB patients [11,12,13,14] as compared to healthy individuals. These contrasting results may be a consequence of analyzing γδ T cells from patients at different stages of disease progression or different subsets of γδ T cells (i.e., total γ T cells or their Vδ1 or Vδ2 subsets); accordingly, decreased levels of circulating Vδ2 T cells were correlated with more severe pulmonary lesions in acute pulmonary TB patients [15,16], which were defective in IFN-γ production both in adult and in pediatric ATB, as compared with TBI subjects and HD [17,18].

In the present study, we hypothesized that distinct subsets of γδ T cells are associated with either protection against or disease progression in TB. To achieve this, we have utilized large-scale integration of scRNA-seq and bulk RNA-seq data to identify the phenotypic and molecular features of peripheral blood γδ T cells. Our findings reveal a significant expansion of γδ T cells in TBI subjects, marked by elevated CD81 expression. This study offers new insights into the potential role of γδ T cells in protective immune responses against *M. tuberculosis*.

## 2. Materials and Methods

### 2.1. Sample Collection

The study included participants with ATB, TBI, and HD. The diagnosis of ATB was established based on clinical symptoms, chest radiography, microscopy for acid-fast bacilli (AFB), sputum culture for *M. tuberculosis*, and response to anti-tuberculosis (TB) treatment. In contrast, individuals with TBI were identified by a positive QuantiFERON-TB Gold Plus test but showed no clinical symptoms or radiologic signs of ATB. Participants with HIV or other immunosuppressive conditions were excluded. The enrolled subjects were tested before the administration of the anti-tubercular therapy or prophylaxis.

A total of 15 participants were enrolled: 5 with ATB, 5 with TBI, and 5 HD. Each participant provided 6 mL of blood in an EDTA tube prior to the initiation of anti-TB or TB preventive therapy. The blood samples were promptly processed to isolate peripheral blood mononuclear cells (PBMCs), which were then stored at −197 °C to preserve their integrity until analysis.

### 2.2. Data Collection

All datasets analyzed in this study were obtained from the National Center for Biotechnology Information’s (NCBI) Gene Expression Omnibus (GEO) database (https://www.ncbi.nlm.nih.gov/geo/, accessed on 21 October 2023), a publicly accessible repository for gene expression data. A search of GEO profiles related to TB and TBI was conducted using the terms “Tuberculosis” [MeSH Terms] OR “active tuberculosis” [All Fields] AND “Homo sapiens” [Organism], which resulted in the identification of 12 distinct studies (GSE37250, GSE39939, GSE39940, GSE40553, GSE42825, GSE42826, GSE42827, GSE42830, GSE42831, GSE42832, GSE83456, and GSEBruno [19]). To ensure a focused and consistent analysis, only the Platform-GPL10558 Illumina Microarray was used to minimize batch effects. Peripheral whole blood samples were specifically selected as the primary biological material to investigate differential gene expression profiles across various conditions (Supplementary Figure S1).

### 2.3. Data Processing and Differential Gene Expression Analysis

Differentially expressed genes (DEGs) across various TB conditions were identified using the R package DESeq2 v1.38.2, which enabled robust differential gene expression analysis on bulk RNA-seq data from different conditions. The RNA-seq counts were normalized, and a variance stabilizing transformation (VST) was applied to prepare the data for downstream analysis. Differential expression analysis was conducted using the default Wald test in DESeq2, with *p*-values adjusted via the Benjamini–Hochberg method. Genes with an adjusted *p*-value < 0.05 and an absolute fold change > 1 were classified as DEGs. Log-transformed data were computed with DESeq2, and batch effects were removed using the R package limma v3.44.3. After batch correction, the data underwent principal component analysis (PCA) and weighted correlation network analysis (WGCNA) (Supplementary Figure S2). This comprehensive strategy ensured the accurate identification of genes with significant expression changes across different TB conditions, laying the groundwork for further analysis. To visualize the results, a volcano plot illustrating the relationship between fold change and statistical significance was created. Additionally, a heatmap generated with the ComplexHeatmap R package provided an overview of gene expression across conditions. The top upregulated genes were clustered by Euclidean distance, and each gene cluster underwent enrichment analysis using MSigDB 2023 gene sets. The findings were displayed via a scatterplot showing the odds ratio (*x*-axis) and −log10 (*p*-value) (*y*-axis). The entire analysis, including the creation and interpretation of visualizations such as volcano plots and heatmaps, was performed using the latest version of R.

### 2.4. Reference-Based Decomposition

The Bisque R toolkit was used for reference-based decomposition to precisely estimate cell composition from bulk expression data using a single-cell reference. This method leverages single-cell data for bulk expression decomposition, employing a regression-based approach with scRNA-seq or snRNA-seq data. Bisque creates a reference expression profile and learns gene-specific transformations in bulk expression, allowing for robust RNA-seq data decomposition. To improve the accuracy of our analysis, we incorporated a newly developed single-cell reference of human peripheral blood specifically created for this study.

### 2.5. Peripheral Immune Cell and γδ T Cell Reference Map

scRNA-seq data from 30 different studies encompassing 100 samples and a total of 160,000 high-quality cells were integrated (see Supplementary Figure S3). Analyses were conducted using Seurat (Version 4.3.0) and SingleR (Version 2.0.0) [20]. Quality control was assessed based on the number of detected feature genes and the percentage of mitochondrial gene expression. To ensure accurate identification of different immune cell subsets, cells with over 1000 detected genes and less than 10% mitochondrial gene expression were deemed high-quality, while those with more than 10% mitochondrial gene expression were excluded. The samples that did not meet the criteria have been omitted from this reference map. Gene counts were normalized using Seurat’s normalize data function, and all cells were integrated using RPCA integration. The integrated data from all samples were clustered with 50 principal components and visualized using uniform manifold approximation and projection (UMAP). Cell type annotation was carried out using ScType [21], and cells identified as γδ T cells were selected for further analysis. The Seurat package was employed to determine the feature genes of γδ T cell subsets, and a single-cell atlas of peripheral γδ T cells was used as a reference to estimate cell composition from bulk expression data with single-cell precision.

### 2.6. Preparation of PBMCs

PBMCs, previously isolated from blood samples using a Ficoll-Paque density gradient centrifugation method in EDTA-coated tubes and stored in liquid nitrogen, were thawed, washed, and resuspended in RPMI 1640 medium supplemented with 10% FBS and antibiotics (penicillin at 100 U/mL and streptomycin at 100 μg/mL, all from Sigma-Aldrich, Saint Louis, MO, USA). The PBMCs were counted using Trypan blue, transferred to flow cytometry tubes, and then washed with 1 mL of BD staining buffer.

### 2.7. Staining of Surface Antigens for Flow Cytometry

PBMCs (10^6^ cells) were aliquoted into flow cytometry tubes, stained before with Zombie Aqua™ Fixable Viability Kit, and then with monoclonal antibodies (mAbs) to CD3 (APC-H7-conjugated, clone SK7, BD Pharmingen^TM^, BD Bioscience, San Jose, CA, USA), TCR-γδ (PE-conjugated, clone REA591, Miltenyi Biotec, Bologna, Italy), CD81 (APC-conjugated, clone REA513, Miltenyi Biotec, Bologna, Italy), CD27 (PE-Vio770-conjugated, clone REA499, Miltenyi Biotec., Bologna, Italy), CD45RA (BV605-conjugated, BD Bioscience, San Jose, CA, USA). After incubating for 30 min in the dark at room temperature, the cells were washed twice with 1 mL of Staining Buffer (PBS without Ca^2+^ and Mg^2+^, 1% FBS, 0.09% sodium azide) and resuspended in 300 µL of Staining Buffer before being analyzed using flow cytometry. Samples were run on a BD FACS Lyric^TM^ flow cytometer, and data were evaluated with BD FACSuite™ V1.5 Application (BD Biosciences, San Jose, CA, USA) after collecting 100,000 gated events (lymphocytes). Peripheral blood lymphocytes were gated using forward (FSC) and side scatter (SSC) parameters, single cells and live cells, T γδ^+^ CD81^+^ cells were identified as CD3-positive, TCR γδ-positive, CD81-positive. Expression levels of CD81 were evaluated based on mean fluorescence intensity (MFI) values. Relevant isotype controls were also used. Samples were analyzed by FlowJo software (v10.10 Treestar Inc. Ashland, OR, USA).

### 2.8. Computational Analysis of Flow Cytometry Data

FlowCT was utilized to analyze the flow cytometry dataset. Automated clustering was carried out with FlowSOM, which employs self-organizing maps to group cells with similar expression profiles into clusters. The cluster annotations were derived by visualizing the expression levels of each marker using uniform manifold approximation and projection (UMAP). Additionally, γδ T cells were further subclustered through phenotyping via accelerated refined community partitioning.

### 2.9. Statistical Analysis

Statistical analysis was conducted using R software version 4.0.3 and GraphPad Prism version 9.1. To compare continuous variables between the two groups, we employed the Wilcoxon test. For comparisons involving three or more groups, we used the Kruskal–Wallis test. Statistical significance was determined with a *p*-value threshold of less than 0.05. Data analysis and visualization were performed with the R packages ggplot2, ggstatsplot, and ggpubr [22].

## 3. Results

### 3.1. DEG and GSEA Analysis Reveal Enrichment of the CD81-Dependent TCR Signaling Pathway in TBI Subjects

**Figure 1:**
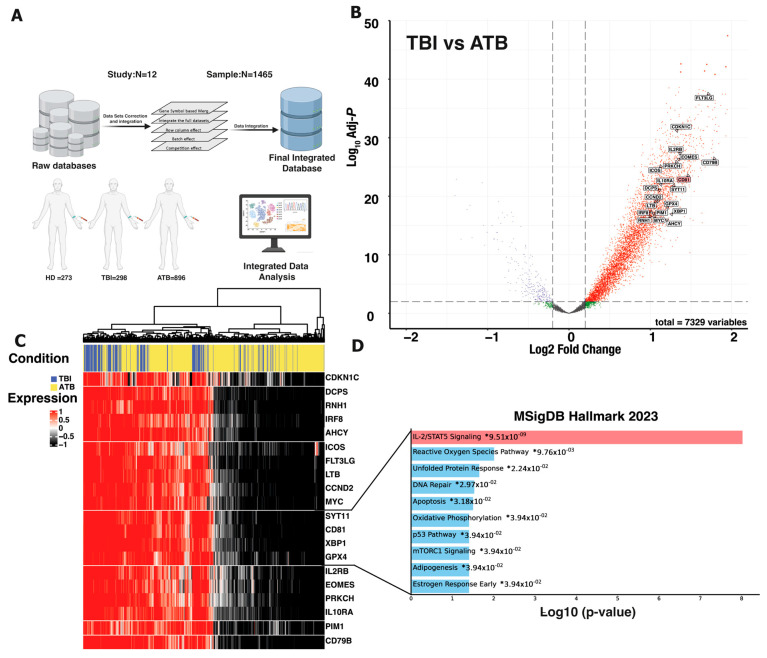
Integration of RNA-seq data and gene expression analysis reveals γδ T cell activity in TB. (**A**) Comprehensive RNA-seq datasets include HD with *n* = 273, TBI with *n* = 298, and ATB with *n* = 896. (**B**) Volcano plots display gene expression differences between TBI and ATB samples. Each point represents a gene, with the *x*-axis showing log2 fold change and the *y*-axis representing −log10 *p*-value. Red points denote genes significantly upregulated in TBI compared to ATB, while blue points highlight genes significantly downregulated. Black points represent genes with no significant expression changes. TCR signaling genes with notable regulation (≥0.2-fold change, *p*-value ≤ 0.001) are emphasized, focusing on upregulated genes associated with the γδ T cell signature. (**C**) A heatmap of TCR signaling-related genes reveals distinct clusters of genes that are significantly higher in TBI compared to ATB. (**D**) Enrichment analysis of hallmark gene sets shows significant shared pathways, illustrated in a heatmap gene cluster. Each point represents a gene set (*x*-axis) and −log10 (*p*-value) (*y*-axis). IL2/STAT5 signaling emerges as the most significantly enriched pathway in TBI versus ATB conditions.

As peripheral blood from individuals with varying *M. tuberculosis* infectious statuses may show different transcription profiles, we examined gene expression and functional enrichment in the peripheral blood of TB patients and TBI subjects, before therapy or prophylaxis administration, using bulk RNA-seq data from 1467 samples (TB = 896, TBI = 298, HD = 273). Importantly, 645 samples from HIV-positive individuals were excluded from the analysis, leaving a final dataset of 1467. After normalization and integration of different studies we used the Gene Ontology Biological Processing (GO) gene set in combination with differential gene expression analysis (Figure 1A). We calculated the fold change in gene expression levels, and pairwise comparisons between TBI and TB condition were made using the ratios of the fold changes. The results of differential gene expression analysis between peripheral blood samples from TB and TBI subjects are shown in Figure 1B as a volcano plot. We were interested as to whether TCR costimulatory signaling was elevated in TBI as compared to TB conditions. The results obtained reveal that there are notable variations in the expression of many genes between the TBI and TB samples. Specifically, the genes marked on the plot—for example *IL2RB*, *ICOS*, *LTB*, *GPX4*, and *CD79B*—are markedly elevated in TBI samples. These genes have been linked to the TCR signaling pathway (KEGG = hsa04660) [23], indicating a potential critical function for this pathway in the immunological response against *M. tuberculosis* (Figure 1C). It is interesting to note that there is a distinct difference between TBI and TB conditions in the TCR signaling pathway gene expression. In contrast, significant contemporaneous alterations were observed in the genes *SYT11*, *CD81*, *XBP1*, and *GPX4*, which have an association to the IL-2-STAT5 signaling pathway. (*p*-value = 9.51 × 10^−9^) (Figure 1D).

### 3.2. Immune Cell Composition in Various TB Conditions by Virtual Deep Immunoprofiling

**Figure 2:**
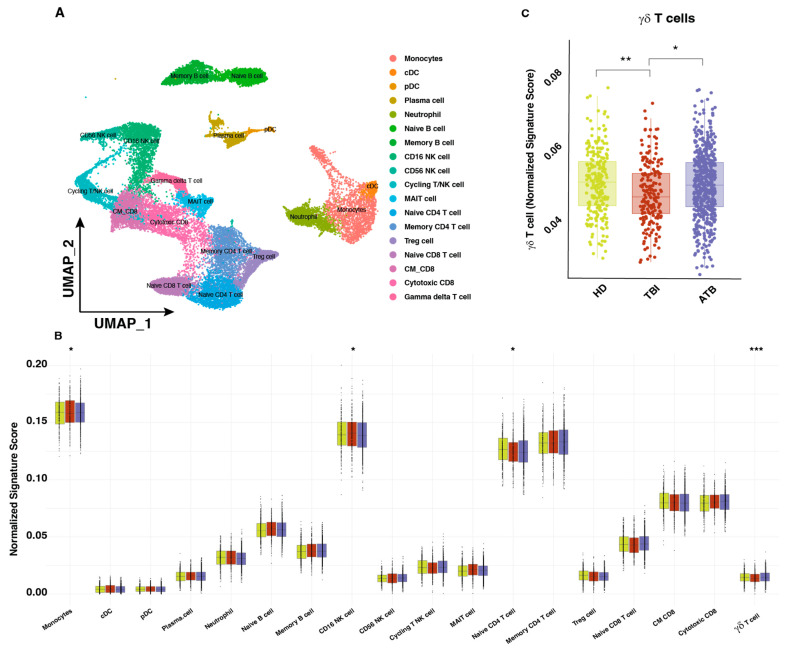
Virtual single-cell RNA-seq shows changes in total and γδ T cells in different TB conditions. (**A**) Following normalization and dimensional reduction, 160 K cells from 30 separate investigations involving 100 samples were combined, resulting in an identification of 8 distinct clusters. (**B**) Different cell subset change based on normalized signature result using PBMC reference map for bulk expression deconvolution showed significant change in CD16^+^NK, naive CD4^+^T cell, and γδT cell. (**C**) Focusing on γδ T cell subset showed significant change in TBI [25]. A two-way *t*-test was used to determine statistical significance, and *p*-values are shown by the symbols (*p* *** ≤ 0.001, *p* ** ≤ 0.01, *p* * < 0.05).

We conducted a virtual deep immunoprofiling analysis to gain a better understanding of immune cell type changes across various TB conditions. A comprehensive scRNA-seq reference panel comprising information from 160,000 cells in 30 studies with 100 subjects was assembled from 110 samples. We used UMAP to clusterize all PBMCs into two-dimensional space and identify different cell types utilizing this large-scale single cell map. Eighteen clusters divided in eight main cell types were found using unsupervised clustering and marker gene analysis (αβ T cells, B cells, NK cells, monocytes, neutrophils, MAIT cells, and γδ T cells) (Figure 2A). This reference map facilitated the application of an in-house developed algorithm for cell deconvolution in bulk RNA-seq data from our integrated dataset, covering our clinical groups and 1467 samples (HD = 273, TBI = 298, TB = 896), excluding the HIV patient cohort as previously described. We removed batch effects to guarantee reliable results. As previously reported [24], γδ T cells across all T cell subsets displayed a significant decrease in TBI subjects, as compared to HD and ATB patients (Figure 2B). This suggests that a discrete population of γδ T cells could function as a biomarker to differentiate between ATB and TBI condition. To achieve this, we conducted an in-depth analysis of γδ T cells.

### 3.3. Crosslinking of CD81 and γδ TCR via PIP3 Activates AKT Signaling Enriched in TBI

**Figure 3:**
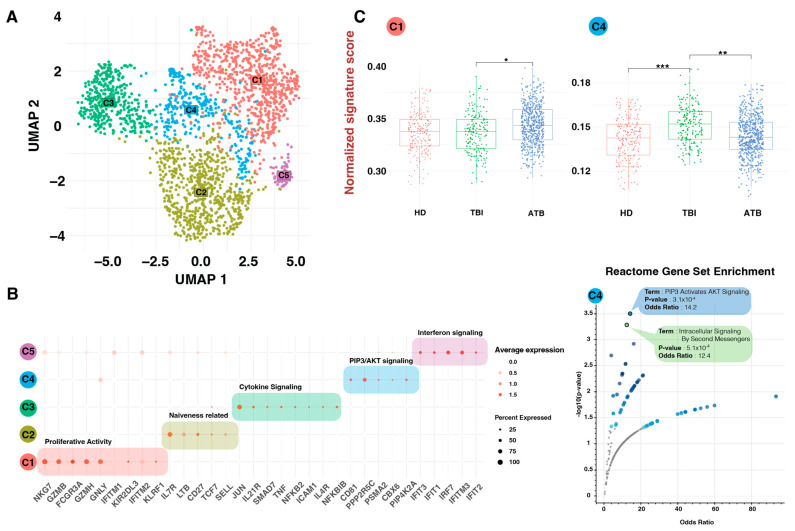
Dynamic changes in γδ T cell subsets in different TB conditions. (**A**) Dimensional reduction in virtually sorted γδ T cells demonstrate 5 different clusters based on the different enriched signal. (**B**) Dotplot of the top conserved markers expressed in each γδ T cell type (left) and enrichment analysis of C4 cluster conserved markers. (**C**) Bulk expression of data decomposition reveals statistically significant changes in γδ T cell C4 cluster. The *p*-values, calculated through a two-way *t*-test, are represented by symbols (*p* *** ≤ 0.001, ** ≤ 0.01, * ≤ 0.05).

We virtually sorted γδ T cells from the original reference maps to perform an in-depth comprehensive analysis. We re-clustered about 1467 high quality γδ T cells and performed new dimensional reduction which led to the identification of five unique γδ T subclusters (Figure 3A), using conserved marker of each cluster and enrichment analysis on conserved marker (Figure 3B). We determined the most significant signal for each cluster by utilizing the top 20 highly conserved markers based on an adjusted *p*-value < 0.001 and fold change > 1. This method improved the validity of our results by enabling us to identify certain markers that were consistently expressed throughout several clusters. By enrichment on these conserved markers, we were able to distinguish between clusters with accuracy and determine the distinctive characteristics of each cluster, leading to a greater understanding of the molecular signatures associated with each γδ T cell subset (Figure 3B). Cluster C1 was enriched for genes linked to proliferative activity such as *NKG7*, *GZMB*, *FCGR3A*, and *GZMH*. Cluster C2 exhibited markers indicative of a naive state, including *SELL*, *TCF7*, *LTB*, and *IL7R*. Cluster C3 was notable for elevated cytokine signaling, particularly *TNF* and *IL4R*. Cluster C4 was characterized by high *CD81* expression as a conserved marker, and Cluster C5 was enriched for interferon signaling, as evidenced by the expression of highly conserved markers such as *IFIT1*, *IFIT2*, *IFIT3*, and *IRF7*. Amongst the five γδ T cell clusters, we identified a unique cluster (C4) that displayed significant enrichment of TCR signaling pathway via “PIP3 activates AKT” and “Intracellular Signaling by Second Messengers”. The result of enrichment indicated these signaling pathways, combined with CD81 expression, were highly enriched in this cluster, with a *p*-value of less than 0.001 and an odds ratio greater than 10 (Figure 3B).

The distribution of the five γδ T cell subtypes among the different TB condition was next examined. As anticipated from the bulk RNA-seq results, Cluster C4 (CD81^+^) showed increased abundance in TBI samples, based on subset estimation using cell type deconvolution (Figure 3C). This distinct molecular signature suggests a specialized role for this cluster in immune signaling and regulation in different TB conditions.

**Figure 4:**
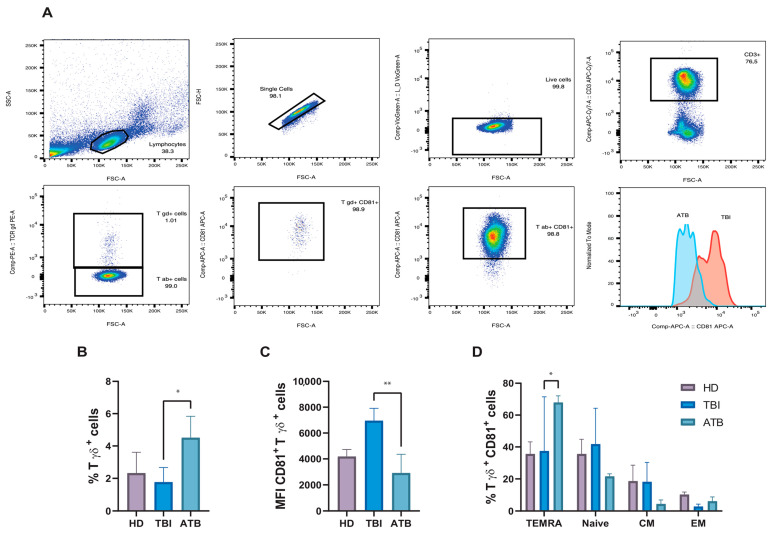
Flow cytometry analysis of γδ T cell subsets in peripheral blood of ATB patients (*n* = 5), TBI subjects (*n* = 5) and HD (*n* = 5). (**A**) Gating strategy to access γδ T cell subsets: lymphocytes were gated using forward (FSC) and side scatter (SSC) parameters, single cells, and live cells. γδ T cells were identified as CD3-positive, TCR γδ-positive cells, the histogram represents the MFI of CD81 expression in ATB (sky blue) and TBI (orange). (**B**) Flow cytometry analysis of total γδ T cells and (**C**) MFI of CD81 expression by γδ T cells in PBMC of HD, TBI subjects, and ATB patients. (**D**) Separation of CD81^bright^ γδ^+^ T cell phenotypes based on expression of CD45RA^+^ CD27^−^ (terminally differentiated, TEMRA), CD45RA^+^ CD27^+^ (Naive), CD45RA^−^ CD27^+^ (central memory, CM), CD45RA^−^ CD27^−^ (effector memory, EM); bars represent mean with SD values. *p*-values were calculated using the Kruskal–Wallis test, including multiple test correction (* *p* ≤ 0.05; ** *p* ≤ 0.005).

To validate our in silico results, we applied flow cytometry analysis to study peripheral blood γδ T cells across the different groups. Figure 4A shows the FACS gating strategy of one representative sample, while Figure 4B shows the frequency of γδ T cells from HD, TBI subjects, and ATB patients. As shown in Figure 4B, γδ T cells, identified and described under Materials and Methods, are significantly increased in ATB patients, as compared to TBI. Most interestingly (Figure 4C), the MFI values of CD81 expression on γδ T cells were dramatically increased in TBI individuals, as compared to ATB patients, and differences attained statistical significance. In addition, evaluating the expression of CD27 and CD45RA on the CD81^bright^ γδ^+^ T cell population (Figure 4D), a statistically significant increase in the terminally differentiated subset was observed in the ATB patients compared to the TBI individuals. Altogether, the flow cytometry data, although performed on a small number of samples, fully reflect our previous in silico results showing expansion of CD81^+bright^ γδ^+^ T cell subset in TBI.

**Figure 5:**
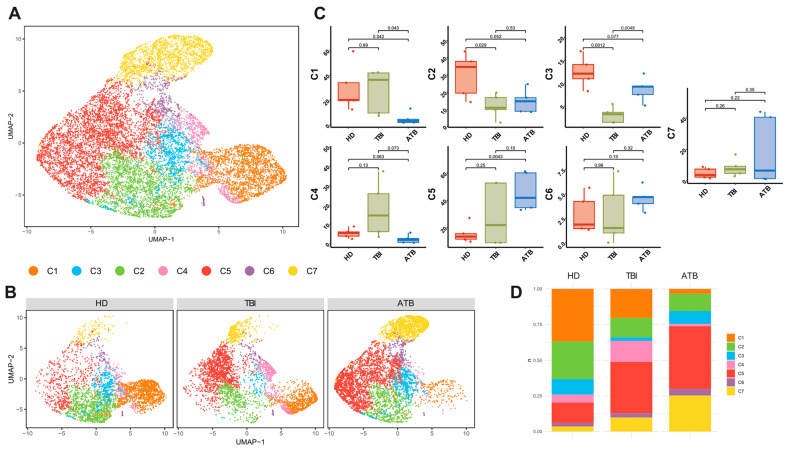
Clustering and identification of γδ T cell subsets. (**A**) Following normalization and dimensionality reduction, γδ T cells were classified into 7 distinct subclusters. An initial clustering process was used to sort all γδ T cells. (**B**) The final clustering of various subsets demonstrated that there was no identifiable batch effect related to the experimental conditions based on cell markers. Initial subset clustering was performed on all cells using FlowSOM. (**C**) γδ T cell clusters, as identified by FlowCT, were evaluated based on experimental conditions. A two-tailed ANOVA test assessed subcluster variations across different conditions, with a *p*-value < 0.05 indicating statistically significant changes. (**D**) The proportion of each subcluster across the three different conditions is illustrated.

To more accurately compare the γδ T cell compartments across different TB conditions, we utilized a semiautomated method called FlowCT [25] to analyze our flow cytometry dataset (see Figure 5). Automated clustering of all live CD3^+^ γδ T cells was conducted using FlowSOM. We visualized the expression levels of each marker through uniform manifold approximation and projection for cluster annotation. Further phenotyping was done using accelerated refined community partitioning to subcluster the γδ T cells.

Our analysis included 100,000 CD3^+^ γδ T cells from HD (*n* = 5), 80,000 CD3^+^ γδ T cells from individuals with TBI (*n* = 5), and 70,000 CD3^+^ γδ T cells from ATB (*n* = 5). These samples were combined into a single integrated dataset. Unsupervised clustering of this dataset, comprising 250,000 CD3^+^ γδ T cells, identified seven distinct clusters (see Figure 5A). Four of these clusters corresponded to previously identified CD3^+^ γδ T cell subsets from classic FACS analysis (C1 = CM, C2 = EM, C6 = Naive, C7 = TEMRA). Additionally, we discovered three new clusters that do not align with previously described CD3^+^ γδ T cell phenotypes, likely representing intermediate stages in the γδ T cell differentiation pathway: C3 = transitional naive to CM, C4 = transitional CM to EM, and C5 = transitional EM to TEMRA.

Figure 5B shows the clusters’ distribution in HD, TBI, and ATB; Figure 5C shows the comparison of the different clusters among the different groups while Figure 5D shows the distribution of the clusters for each group.

**Figure 6:**
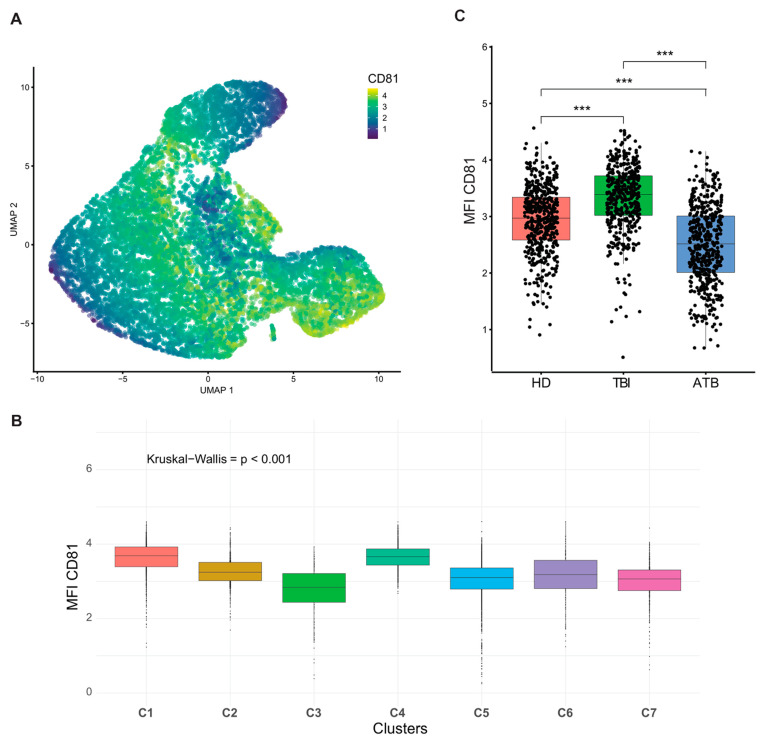
Analysis of CD81 expression by γδ T cell among the groups. (**A**) UMAP density plots of γδ T cells indicate CD81 expression; dark blue shows no/low expression of CD81 and yellow shows high expression of CD81. CD81 MFI in different clusters (**B**) and in different conditions (**C**) is shown. Kruskal–Wallis one-way analysis of log_10_ counts of CD81 between different conditions in total γδ T cells, *** *p* < 0.005.

CD81 was most intensely expressed in all clusters (Figure 6A), with the highest expression detected in clusters C1 and C4 (Figure 6B), but when CD81 expression was compared amongst different conditions, CD81^bright^ γδ T cells were significantly expanded in TBI individuals (Figure 6C).

## 4. Discussion

A better understanding of the role of lymphocytes in immune responses to *M. tuberculosis* is crucial for understanding how they provide overall immune protection. This knowledge will aid in developing new intervention strategies and may distinguish TBI subjects from ATB patients. Such understanding is essential for the global management of TB. Although it has long been known that conventional CD4 and CD8 T cells play an essential role in the immune responses to *M. tuberculosis*, new research suggests that additional non-conventional lymphoid cells may also be involved. Numerous studies have demonstrated the significance of γδ T cells in this regard. One of the first studies to record γδ T cells in a human disease was the γδ cell response in active TB [26].

The finding that γδ T cells expand during *M. tuberculosis* infection in experimental models and in humans has been taken as a proof of their contribution to protective immune responses to TB [9]. Indeed, T cells, and particularly their Vδ2 subset, perform several different functions which are coherent with their anti-mycobacterial activity: they produce cytokines as TNF-α and IFN-γ which play a well-known role in controlling *M. tuberculosis* infection and are also capable of killing macrophages infected with *M. tuberculosis* and reducing the viability of intracellular bacteria [27]. Moreover, they also participate indirectly to protective anti-mycobacterial immune responses by promoting maturation of dendritic cells and providing help to CD8 T cell activation [28,29]. Additional support for the anti-mycobacterial role of γδ cells comes from patients with Mendelian susceptibility to mycobacterial disease: patients with autosomal recessive IL-23R deficiency which suffer mycobacterial disease have a profound failure in *M. tuberculosis*-inducible IFN-γ response in Vδ2 T cells [30]. Similarly, leukocytes from RORγ- and RORγ T-deficient individuals also displayed an impaired IFN-γ response to *M. tuberculosis* that principally reflected profoundly defective IFN-γ production by circulating γδ T cells [31].

We gained interest in investigating the composition of different γδ T subclusters in the three examined groups because changes in the frequency of circulating lymphocytes have not previously been exclusively linked to TBI status. Here, γδT cell subcluster distribution across different tested groups revealed that latent *M. tuberculosis* infection leads to the expansion of a unique type of CD81^bright^ γδ T cells (Figure 3C), and skews them toward an AKT signaling, which is generated by PI3K and involves cell activation via the cellular second messenger PIP3. The PI3K/AKT pathway is a crucial signaling cascade involved in the regulation of cell growth, survival, and metabolism. Tyrosine kinase-associated receptors, such as the T cell receptor (TCR), co-stimulatory (like CD28 and CD81), and cytokine receptors, are the main sources of class IA PI3K activation in CD8 T cells. PI3K/Akt signaling pathway in CD8 T cells is stimulated by signaling triggered by exposure to IL-12 and common gamma chain (γ_c_) receptor-related cytokines such as IL-2, IL-7, IL-15, and IL-21 [32]. Among the γc cytokines, IL-2 generates high and persistent PIP3 levels, whereas IL-15 stimulates PI3K comparatively weakly and produces low PIP3 levels [33]. In the present study, we demonstrate a presence of a unique CD81^bright^ γδ T cell subset which altered in TBI subjects and most notably revealed an enrichment of the PI3K/Akt signaling pathway (Figure 3B). Conversely, the cluster of γδ T cells (C1) exhibited opposite behavior, with a significant increase in active TB individuals compared to other groups. This cluster, which demonstrated high proliferative activity, expressed GZMB, GZMH, and GNLY as conserved markers (Figure 3B). These findings align with previous phenotypic and molecular studies, which reported an elevation of GZMB in both HIV-negative and HIV-positive TB patients, indicating its potential as a diagnostic marker [34]. Additionally, GZMA, internalized by mycobacteria-infected cells, inhibits the growth of intracellular mycobacteria, which increases under TB conditions but does not significantly affect the control of *M. tuberculosis* infection in a mouse experimental model [35].

Two distinct signals are needed to activate naive T cells; the TCR’s interaction with the antigen and the second signal, known as costimulatory, rather than the other most well investigated T-cell costimulatory molecule such as CD28, CD81 is less investigated, but it is clear that CD81 and the PI3K/AKT pathway play crucial protective roles in T cell responses during human infections. CD81 facilitates T cell activation by organizing membrane microdomains and enhancing signal transduction, while the PI3K/AKT pathway ensures T cell survival and function. Together, these mechanisms enable the immune system to mount effective responses against pathogens.

Similarly, and relevant to our study, CD81 and PI3K/AKT pathways play a significant role in the survival and proliferation of T cells during infection including viruses like influenza virus infection. Activation of the PI3K/AKT pathway enhances the survival of T cells by promoting anti-apoptotic signals which are critical for maintaining an effective immune response [36]. The PI3K/AKT pathway also plays a role in the immune response to HPV. Activation of this pathway in T cells enhances their ability to produce cytokines and exert cytotoxic effects against HPV-infected cells. This helps in controlling the infection and preventing the progression of HPV-associated diseases, such as cervical cancer [37]. Moreover, an interesting study on CD4^+^ T lymphocytes in HIV patients demonstrated that CD81 serves as a significant secondary activation signal, particularly for CD4^+^ T cell subsets. In HIV-positive individuals, CD81 expression on CD4^+^ T lymphocytes is notably lower compared to those in healthy donors, compromising the functionality of these lymphocytes. This underscores the critical role of CD81 in T cell response [38].

These examples illustrate the protective roles of CD81 and the PI3K/AKT pathway in T cells during various human infections, highlighting their importance in maintaining effective immune responses. In our research, contrasting with findings in HIV patients, we observed a significant increase in CD81 expression in γδ T cells of individuals with latent infections. Conversely, patients with active disease exhibit CD81 expression on γδ T cells like that of healthy donors. The heightened expression of CD81 on γδ T cells in our study could be associated to the enhanced activation capacity of these lymphocytes due to the robust costimulatory signal from CD81 engagement. The increased frequency of CD81^bright^ γδ T cells in TBI individuals could be the result of continuous exposure to *M. tuberculosis* antigens, suggesting that this cell subset might be somehow involved in the control of *M. tuberculosis* infection at a latency stage and *M. tuberculosis* reactivation. The IL-2/STAT5 pathway is closely interconnected with the TCR signaling pathway and plays a critical role in modulating its strength and outcome. IL-2 signaling can amplify TCR-induced proliferation, survival, and function of T cells. Conversely, TCR activation upregulates the expression of IL-2 and its receptor components, establishing a feedback loop that sustains T cell activation and enhances their response [39]. These pathways are not distinct but rather integral to a coordinated immune response, ensuring that T cells can effectively respond to pathogens through TCR signaling. Thus, the mention of the IL-2/STAT5 pathway in the results aligns with the broader context of the role of TCR signaling in immune modulation.

Recently, a study showed the expansion of a distinctive subset of NK-like CD8^+^ γδ T cells (predominantly Vδ1) in TBI subjects, but it was not reported whether this subset expresses CD81 [40].

## 5. Conclusions

Although this study is limited by the relatively small sample size analyzed phenotypically (i.e., via flow cytometry), it offers significant value as it represents the first effort to integrate scRNA-seq and bulk transcriptomics on extensive datasets to identify alterations in immune cell composition in human TB. This approach, which has recently proven useful in cancer research [41,42], could open new avenues for evaluating biomarkers and potential correlates of protection in human TB.

This indicates that targeting Vδ2^+^ T cells could be a promising approach for developing TB vaccines or immunotherapies. For instance, administering PAgs alongside IL-2, which promotes the expansion of the Vδ2^+^ subset, has been shown to improve TB treatment outcomes in macaques [43]. Additionally, a recent clinical trial using allogeneic Vδ2^+^ T cell therapy for MDR-TB demonstrated a reduction in *M. tuberculosis* levels and improvement in pulmonary lesions, suggesting an enhancement of the host’s immune response [44].

## Data Availability

The raw data generated from both bulk and single-cell RNA sequencing are publicly available and all script and row cytometry data presented in this study are available upon request from the corresponding author.

## Supplementary Materials

The following supporting information can be downloaded at: https://www.mdpi.com/article/10.3390/cells13181529/s1, Figure S1 Flowchart of the processing steps for the final TB bulk RNA-seq datasets, Figure S2 Effect of normalization and batch effect correction on different datasets, Figure S3 integration of 100 samples from 30 studies, encompassing PBMCs from both healthy subjects and subjects with different diseases, to create a unified cell reference.
