# Supplementary material for: Deep Immunoprofiling of Large-Scale Tuberculosis Dataset at Single Cell Resolution Reveals a CD81bright γδ T Cell Population Associated with Latency
Source: Cells. 2024 Sep 12;13(18):1529. doi: 10.3390/cells13181529 (PMC11430301; doi:10.3390/cells13181529)
Supplement: Supplementary file 1 [file cells-13-01529-s001.zip › cells-3129089-supplementary.pdf]

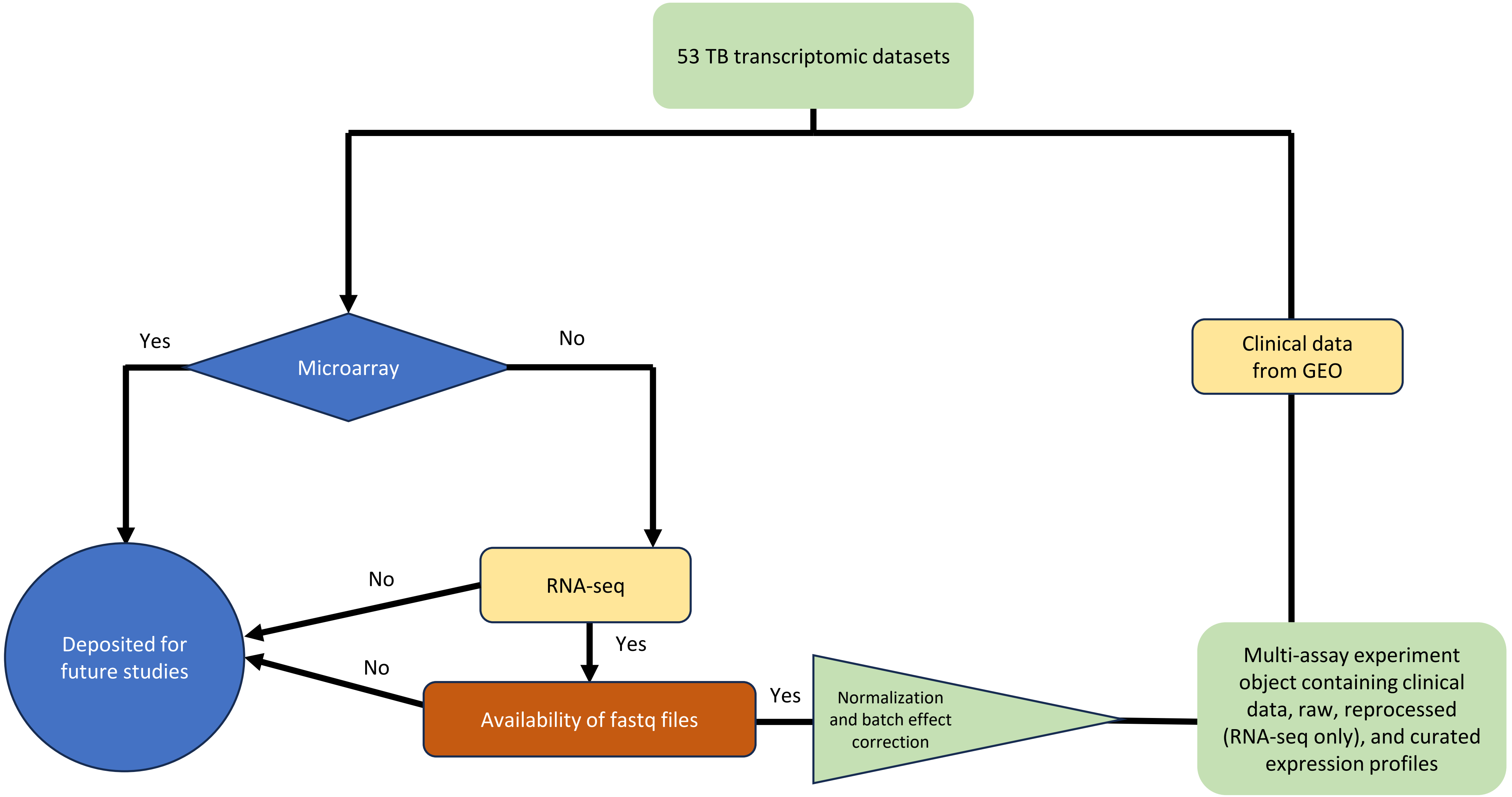

Supplementary Figure S1 . Flowchart of the processing steps for the final TB bulk RNA-seq datasets. Raw FASTQ files were aligned to the GRCh38 genome, followed by normalization of the final count matrix and batch effect correction to produce a fully integrated dataset.

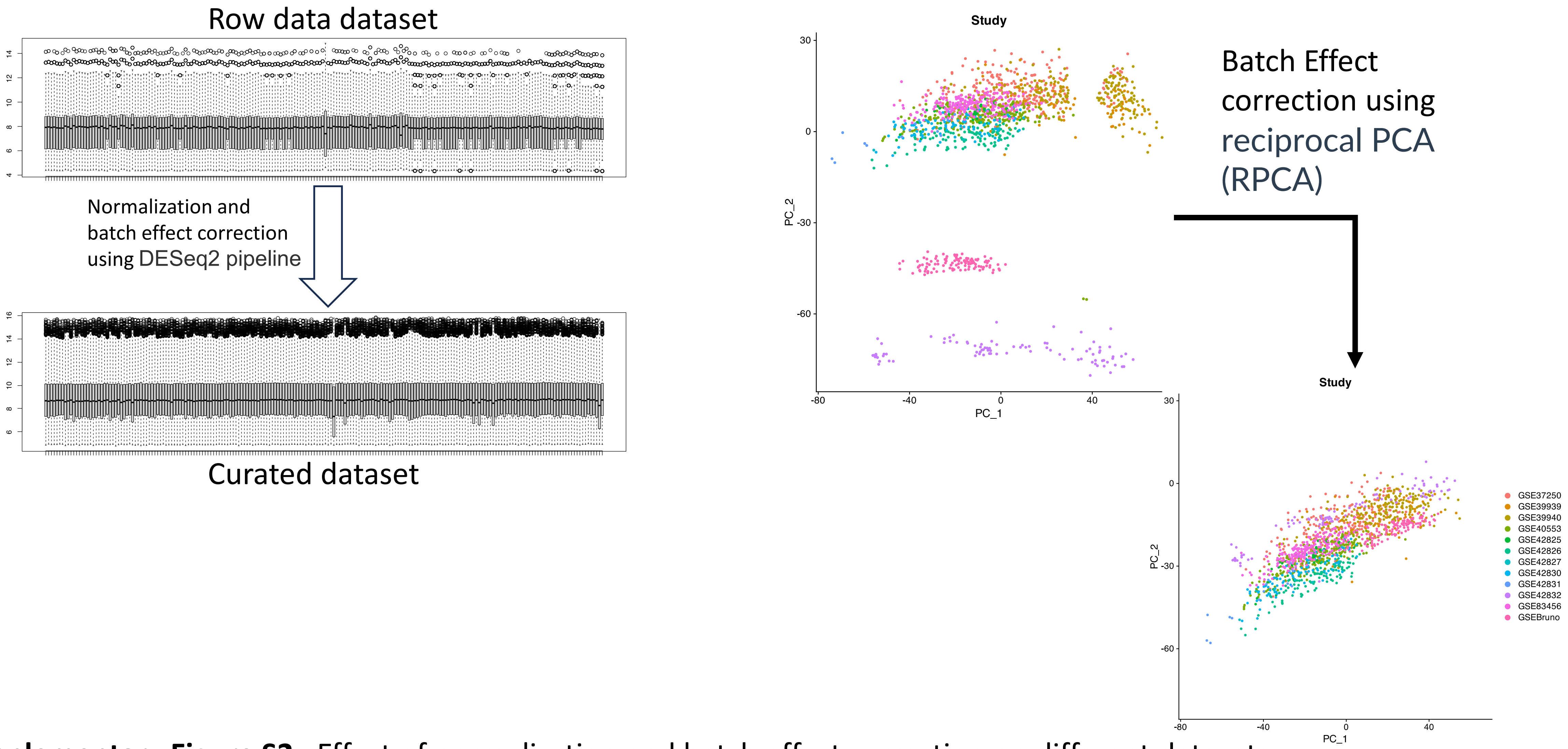

Supplementary Figure S2 . Effect of normalization and batch effect correction on different datasets.

Integrated map characters

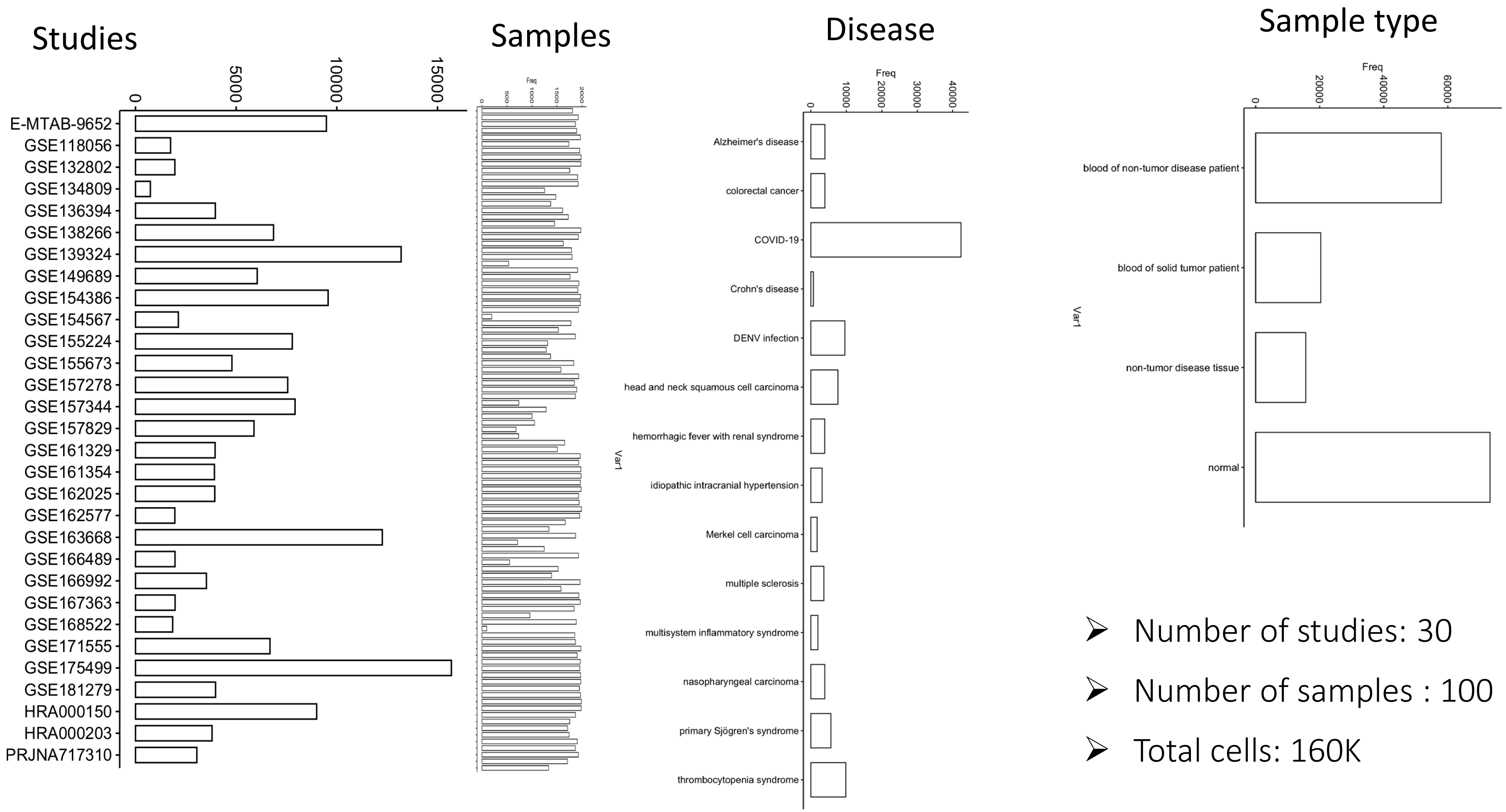

- Number of studies: 30
- Number of samples : 100
- Total cells: 160K

Supplementary Figure S3 : integration of 100 samples from 30 studies, encompassing PBMCs from both healthy subjects and subjects with different diseases, to create a unified cell reference. This reference includes both the standard signatures of cell subsets and the altered cellular signals within the same subsets in various diseases. This dataset was utilized for independent component analysis (ICA) and deconvolution of known cell subsets in bulk RNA-seq.
